# Cronassial Ameliorates Autoimmune Encephalomyelitis by Inhibiting Lipid Oxidation and Carbonyl Stress in the Brain and Spinal Cord of Rats

**DOI:** 10.1155/2023/5552740

**Published:** 2023-11-10

**Authors:** Gayane Ghazaryan, Hasmik Zanginyan, Laura Hovsepyan, Artyom Azatyan, Maria Ghazaryan, Lusine Mardanyan

**Affiliations:** ^1^Institute of Molecular Biology NAS RA, Laboratory Experimental Biology, Yerevan, Armenia; ^2^Davidyants Laboratories, 10, 3 Sasna Tzrer St, Yerevan 0054, Armenia

## Abstract

In recent years, the pathogenetic role of oxidative stress in damaging myelin cells, a precursor to the development of myelin-related diseases such as multiple sclerosis, has gained increasing significance. Experimental autoimmune encephalomyelitis (EAE) in rats serves as an experimental model for human multiple sclerosis. Our study elucidates and demonstrates the antioxidant properties of Cronassial, a drug containing gangliosides, on the processes of free radical lipid oxidation and oxidative modification of proteins in the brains and spinal cords of rats with EAE. Our research results reveal an elevated production of oxidative stress products, including peroxides, hydroperoxides, and oxidized proteins, in experimental animals. This phenomenon is one of the factors contributing to myelin damage. Administering a ganglioside-containing drug normalizes the consequences of oxidative stress and inhibits the formation of reactive oxygen species. Consequently, the data obtained highlight the neuroprotective and antioxidant effects of Cronassial when administered to animals with autoimmune encephalomyelitis.

## 1. Introduction

Diseases whose main manifestation is the destruction of myelin are among the most significant challenges in clinical medicine. The most common disease within this group is multiple sclerosis (MS). Experimental autoimmune encephalomyelitis (EAE) in rats serves as an experimental model for human multiple sclerosis [1, 2]. MS ranks as the fourth most prevalent neurological disease of the Central Nervous System (following acute cerebral circulation disorders, epilepsy, and parkinsonism). It typically begins at a young age and invariably leads to disability.

The pathogenesis of MS involves an inflammatory process, progressive demyelination, and axonal damage. The disease is characterized by the development/progression of numerous sclerotic lesions, primarily in the white matter of the brain and spinal cord, resulting in significant functional impairments. A key diagnostic feature of MS is the presence of demyelination plaques in the brain and spinal cord at various stages of maturation. Clinical manifestations of MS include motor and coordination impairments, sensory deficits, visual disturbances, and cognitive changes in intellect.

The primary immunological phenomena in this disease involve the T-cell immune response to the main protein of myelin. Activated T-cells sensitized to myelin antigens infiltrate the brain, initiating a local inflammatory process [3–5]. Recent data have highlighted the essential role of oxidative stress in myelin cell damage, which is associated with immune inflammation [6]. Accordingly, the use of drugs that reduce oxidative stress development is of particular importance. Literature indicates that gangliosides have the ability to reduce oxidative stress intensity in cells [7]. There is also data regarding the application of gangliosides in cerebral ischemia and stroke [8]. Gangliosides are essential structural components of most eukaryotic cells. They serve as marker lipids of neuronal membranes and glial cells, particularly in synaptic sites, and are key components of neuronal receptors. Gangliosides are involved in various physiological processes in humans and animals, including immune responses, neuronal signal transmission, and regulation of vascular tone, hemostasis, inflammation, and apoptosis. Acting as second messengers, they transmit various external signals into the cell and function as intracellular mediators [8, 9].

## 2. Materials and Methods

### 2.1. Ethics Statement

Adult male outbred rats weighing 200–220 g were provided by the vivarium of the Institute of Molecular Biology of the National Academy of Sciences of the Republic of Armenia. Rats were kept under controlled conditions according to protocol #05072021/1; 07/05/2021, issued by the Committee of the Institute of Molecular Biology of the National Academy of Sciences of the Republic of Armenia for the Care and Maintenance of Animals. Animals were kept at standard temperature, humidity (60–67%), and a 12-hour light/dark cycle. They had free access to food and water.

### 2.2. EAE Induction and Experimental Groups

EAE was induced by immunization with 50 *µ*l of rat spinal cord emulsion (preisolated MBP according to the protocol) + commercial complete Freund's adjuvant (CFA) (SIGMA, F5881) in a ratio of 1 : 2) into the hind paw pads of rats [10].

Rats were divided into groups: control animals; animals with experimental autoimmune encephalomyelitis; animals treated with a ganglioside-containing commercial drug Cronassial@ (Fidia Research Laboratories, Italy); and a group receiving the synthetic drug alpha-tocopherol (vitamin E).

The white matter of the tissue of the brain and spinal cord of experimental rats was used for the study. The animals were decapitated were anesthetized with an intramuscular injection of 20 mg/kg chlorpromazine hydrochloride (chlorpromazine hydrochloride, Sigma-Aldrich, Taufkirchen, Germany) on the 21st day with the development of characteristic signs of the disease. Treatment was started on day 22 and continued for 21 days.

Ganglioside-containing drug Cronassial was administered to animals per os at the rate of 20 mg, of the drug per kg of body weight (20 mg/kg) and for a comparative analysis, the antioxidant alpha-tocopherol was used with the same dosage.

The response of animals to the administration of the drug was assessed by the level of manifestation of neurological deficit and pathomorphological changes in the brain of rats of the experimental groups at the peak of the acute phase of the disease. The following standard indicators were used to assess the clinical severity of the disease, including general factors such as body weight and mortality, as well as indicators of neurological deficit [20].

### 2.3. Sample Preparation

After decapitation, the blood was collected into tubes with Na oxalate and centrifuged for 30 minutes at 1000 × *g*; blood plasma was used.

Homogenization of the brain tissues samples was carried out in an 0.1 M tris-HCl buffer pH 7,2 solution using a glass Potter–Elvehjem homogenizer, placed in ice, under standard conditions. Samples of brain and spinal cord tissues were homogenized in a 0.1 M tris-HCl buffer solution with a pH of 7.2, using a glass Potter–Elvehjem homogenizer placed in ice, under standard conditions. The 10% tissue extracts obtained after centrifugation at 20000 g for 20 minutes were used to determine the levels of malondialdehyde and products of oxidative protein modification.

For histological examination, the brain and spinal cord tissues of animals were fixed in 10% neutral buffered formalin, poured into paraffin, sections 3-4 microns thick were prepared from tissue blocks, stained with hematoxylin and eosin. The stained sections were examined under a light microscope with the possibility of photoregistration.

### 2.4. Determination of Oxidative Modification of Proteins

The oxidative modification of proteins in homogenates of the brain and spinal cord was determined spectrophotometrically based on the quantity of oxidized amino acids [12]. A molar extinction coefficient of 21 × 10^3^ M^−1^ cm^−1^ was used for calculations.

### 2.5. Measurement of Lipid Peroxidation Intensity

Lipid peroxidation intensity was assessed utilizing the malondialdehyde-thiobarbituric acid interaction method, which produces a colored trimethyl complex composed of one molecule of malondialdehyde and two molecules of TBA. The color intensity was quantified spectrophotometrically at a wavelength of 535 nm [13].

### 2.6. Determination of the Content of Diene and Triene Conjugates, Schiff Bases

*Principle of the Method*: This method revolves around determining the content of lipid peroxidation products in the blood by measuring the absorption of a monochromatic light flux in the ultraviolet region of the spectrum using a lipid extract. Optical densities (E) are measured using a spectrophotometer (Evolution 201/220 UV-Visible, ThermoFisher). Each phase is compared to its respective control at wavelengths of 220 nm (for isolated double bond absorption), 232 nm (for DC absorption), 278 nm (for TC absorption), and 400 nm (for SB absorption) [14].

### 2.7. Histopathological Procedures and H&E Staining

After fixing the tissues of the spinal cord and brain of rats in a 10% formalin solution, they were embedded in paraffin for histopathological staining and stained with hematoxylin and eosin. Stained sections were examined under a light microscope ZEISS Primo Star with the possibility of photo registration ZEISS Axiocam 105 color.

### 2.8. Statistical Analysis

For the statistical analysis of the experimental data, the SPSS Statistics 18 package (SPSS Inc., USA) will be used [15]. The nature of the distribution of the data obtained will be determined by the Kolmogorov–Smirnov method. With a nonparametric distribution, a comparative analysis will be carried out using the Mann–Whitney nonparametric test.

## 3. A Study Was Done on the Dynamics of the Weight of Experimental Animals

The condition of the animals was assessed according to a 5-point system (see Table 1).

## 4. Commercial Preparation Cronassial® Composition (Research Laboratories Fidia, Italy)

The active ingredient in Cronassial® (Fidia Research Laboratories, Italy) is a mixture of gangliosides: monosialotetrahexosylganglioside (GM1 21 ± 10%, Mr. 1545), disialotetrahexosylganglioside (GD1a ± 40% and GD1b 16 ± 10%, Mr 1830), trisialotetrahexosylganglioside (GT1b 19 ± 10%, Mr 2127) [16]. The product is a whitish powder, soluble in water but not in organic solvents, stable at room temperature for at least 5 years.

### 4.1. Toxicity of Cronassial® Commercial Preparation (Research Laboratories Fidia, Italy)

The ganglioside mixture Cronassial® does not exert toxic effects. LDx values are high. Gangliosides are typically nonantigenic, as confirmed by negative PCA tests in rats and beagle dogs. There was no evidence of teratogenic effects, as well as no adverse effects on any of the measured reproductive characteristics. Cronassial® does not exhibit detectable toxicity.

## 5. Results

Based on the study of physiological parameters of experimental animals, we observed weight loss in animals with simulated experimental autoimmune encephalomyelitis (EAE) and normalization of these parameters after in vivo administration of a ganglioside-containing preparation and vitamin E (Table 2).

### 5.1. Lipid Peroxidation in Experimental Autoimmune Encephalomyelitis

The main parameters for evaluating free radical oxidation involve the study of lipid breakdown products, resulting in the formation of diene and triene conjugates (DC, TC), hydroperoxides (HPO), Schiff bases (SB), and malondialdehyde (MDA).

According to the results of our study, the content of diene and triene conjugates, Schiff bases, neutral lipids, and phospholipids in the total brain homogenate of experimental animals increased by an average of 2.5–3 times compared to the control group. The amount of MDA in the control group (*μ*mol/g of tissue) was 0.294 ± 0.00001, while in rats with EAE, it was 0.41 ± 1.5.

All parameters of oxidized lipids studied by us also increased in the total homogenate of the spinal cord of experimental animals. DC, TC, and SB in rats with EAE increased on average by 2 times compared to the control, and the amount of MDA in experimental rats increased by approximately 4 times compared to the control (0.08 ± 0.0006 and 0.38 ± 0.01).

After the administration of the ganglioside-containing drug, there is a persistent increase in all parameters we studied compared to animals with EAE; however, they do not reach the control values.

**Figure 1:**
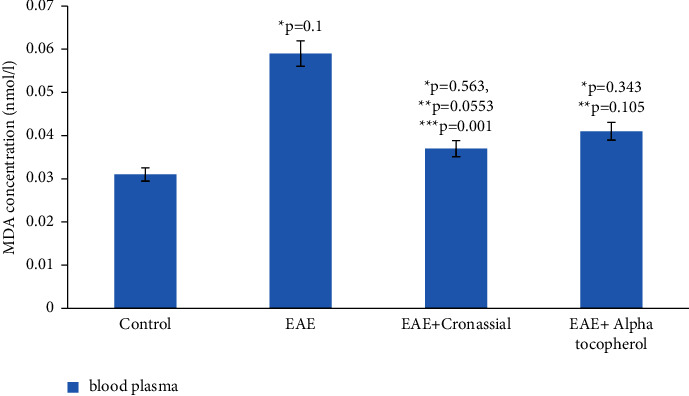
Comparative analysis of the oxidative activity of the commercial drug Cronassial and E307 synthetic alpha-tocopherol (Vitamin E) in blood plasma during EAE. ^*∗*^*p*-compared with control, ^*∗∗*^*p*-compared treatment with pathology. ^*∗∗∗*^*p*-compared with alpha-tocopherol (Vitamin E). In each group, *n* = 6 rats.

**Figure 2:**
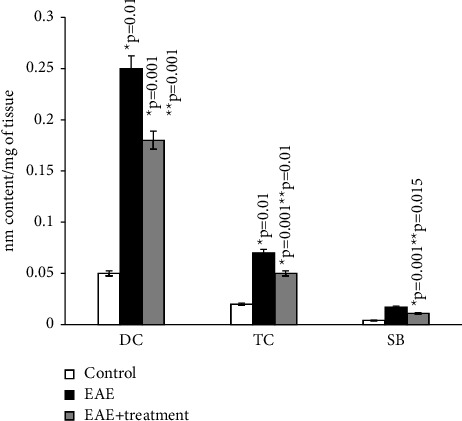
Content of diene (DC), triene conjugates (TC), Schiff bases (SB) in neutral lipids of general homogenate of brain in normal condition and in case of experimental autoimmune encephalomyelitis and in treatment. ^*∗*^*p*-compared with control, ^*∗∗*^*p*-compared treatment with pathology. In each group, *n* = 6 rats.

**Figure 3:**
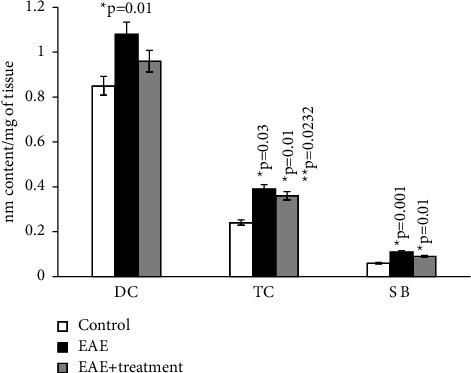
Content of diene (DC), triene conjugates (TC), Schiff bases (SB) in the phospholipids of general homogenate of brain in normal condition and in case of experimental autoimmune encephalomyelitis (EAE) and in treatment. ^*∗*^*p*-compared with control, ^*∗∗*^*p*-compared treatment with pathology. In each group, *n* = 6 rats.

**Figure 4:**
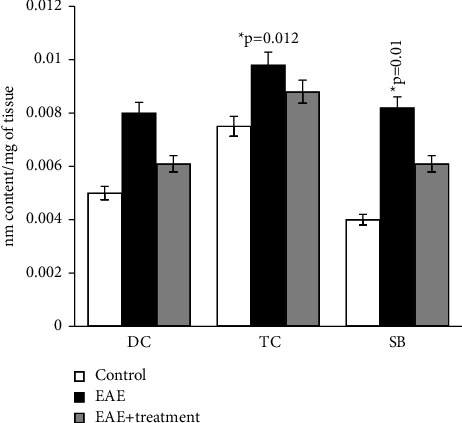
Content of diene (DC), triene conjugates (TC), Schiff bases (SB) in the neutral lipids of general homogenate of spinal cord in normal condition and in case of experimental autoimmune encephalomyelitis and in treatment. ^*∗*^*p*-compared with control, ^*∗∗*^*p*-compared treatment with pathology. In each group, *n* = 6 rats.

**Figure 5:**
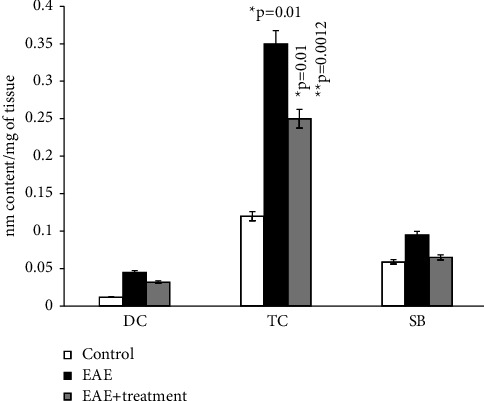
Content of diene (DC), triene conjugates (TC), Schiff bases (SB) in the phospholipids of general homogenate of spinal cord in normal condition and in case of experimental autoimmune encephalomyelitis and in treatment treatment. ^*∗*^*p*-compared with control, ^*∗∗*^*p*-compared treatment with pathology. In each group, *n* = 6 rats.

**Figure 6:**
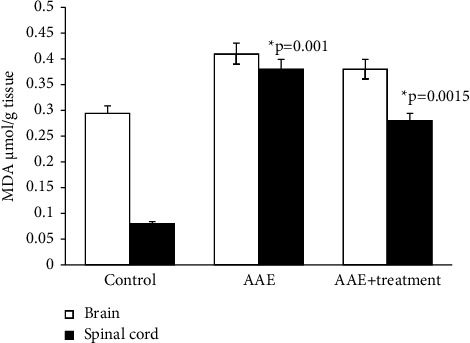
Content of malonic dialdehyde in the homogenates of brain and spinal cord in normal conditions and in case of experimental autoimmune encephalomyelitis and in treatment. ^*∗*^*p*-compared with control, ^*∗∗*^*p*-compared treatment with pathology. In each group, *n* = 6 rats.

**Figure 7:**
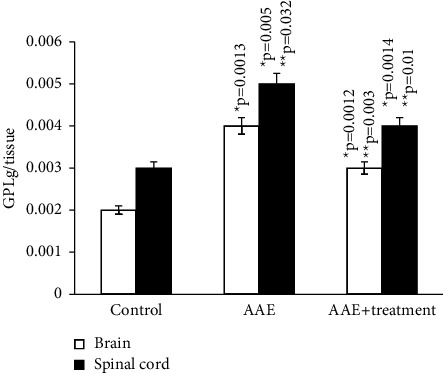
Content of lipid hydroperoxides in the homogenates of brain and spinal cord in normal condition and in case of experimental autoimmune encephalomyelitis and in treatment. ^*∗*^*p*-compared with control, ^*∗∗*^*p*-compared treatment with pathology. In each group, *n* = 6 rats.

As the results of the study show, in the homogenate of the white matter of the brain and spinal cord of rats with simulated autoimmune encephalomyelitis, there is a certain stationary level of intensity of free radical reactions on the 21st day of disease development, and the amount of MDA in the blood plasma of experimental rats increased approximately 2 times compared to the control. (0.059 ± 0.01 and 0.031 ± 0.01) (Figures 1–7).

As a result of our research, we found that during EAE in the blood plasma of experimental animals at equal applied doses (20 mg/kg), Cronassial expresses more active antioxidant properties than alpha-tocopherol (Vitamin E).

### 5.2. Сarbonyl Stress

Reactive oxygen species (ROS) cause oxidative modifications of proteins, leading to membrane damage. Analysis of the obtained data has shown that rats with modeled autoimmune encephalomyelitis, when compared to the control group, have significantly higher levels of protein carbonyl groups in the brain. This indicates an increase in oxidative damage to proteins in cases of autoimmune encephalomyelitis.

**Figure 8:**
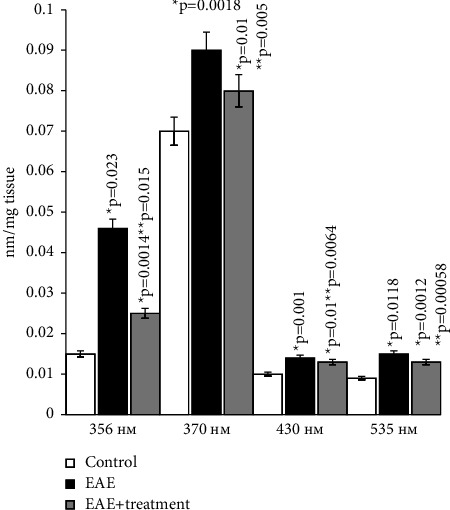
Content of carbonyl derivatives of dinitrophenylhydrazones in brain homogenate in normal conditions and in case of experimental autoimmune encephalomyelitis and in treatment. ^*∗*^*p*-compared with control, ^*∗∗*^*p*-compared treatment with pathology. In each group, *n* = 6 rats. 356 nm-aliphatic ketondinitrophenylhydrazones of neutral nature; 370 nm-aliphatic aldehyddinitrophenylhydrazones of neutral nature; 430 nm-aliphatic ketondinitrophenylhydrazones of alkaline nature; 535 nm-aliphatic aldehyddinitrophenylhydrazones of alkaline nature.

**Figure 9:**
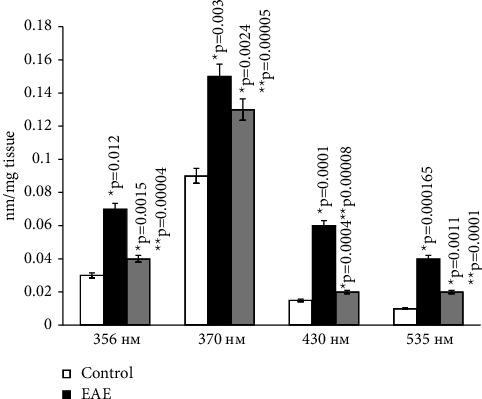
Content carbonyl derivatives of dinitrophenylhydrazones in spinal cord homogenate in normal conditions and in case of experimental autoimmune encephalomyelitis and in treatment. ^*∗*^*p*-compared with control, ^*∗∗*^*p*-compared treatment with pathology. In each group, *n* = 6 rats. 356 nm-aliphatic ketondinitrophenylhydrazones of neutral nature; 370 nm-aliphatic aldehyddinitrophenylhydrazones of neutral nature; 430 nm-aliphatic ketondinitrophenylhydrazones of alkaline nature; 535 nm-aliphatic aldehyddinitrophenylhydrazones of alkaline nature.

The content of ketone and aldehyde derivatives of dinitrophenylhydrazones increased in animals with EAE approximately 2-3 times compared to the control group at all studied wavelengths, both in brain studies and in spinal cord homogenates. Upon oral administration of the ganglioside-containing drug Cronassil to animals, there is a quantitative increase in the above parameters compared to experimental animals, but they do not reach the control values (Figures 8 and 9).

The administration of Cronassial led to a partial normalization of the investigated parameters.

### 5.3. Histopathological Analysis of Samples in EAE

**Figure 10:**
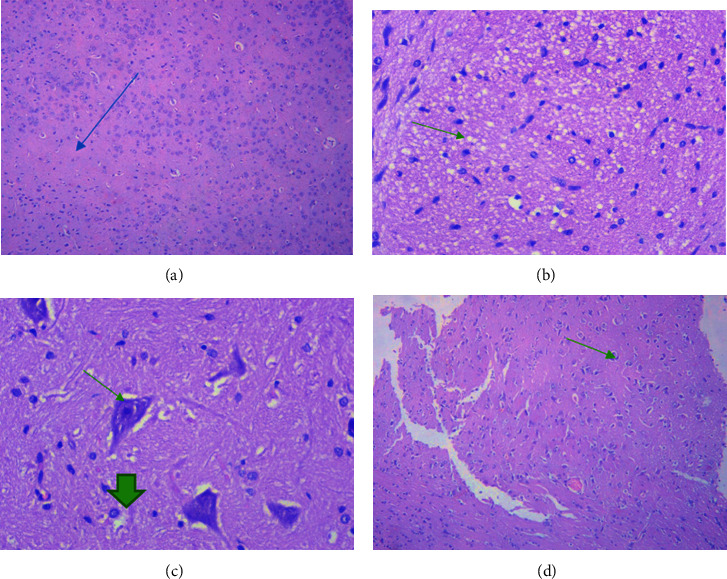
Morphology of rat brain tissue: (a) (control) preserved brain tissue, cortical neurocytes are visible, white matter (arrow) without features. Hemotoxylin, eosin, x40. (b) (EAE) Edema of the white matter of the brain tissue (arrow), punctate focal necrosis, formation of “reticular” tissue. Hematoxylin and eosin, x10. (c) (EAE) Cerebral white matter edema (arrow), pericellular edema in pyramidal (arrow) and oligodendroglial cells (thick arrow). Hematoxylin and eosin, x10. (d) (treatment) predominance of oligodendroglial cells in brain tissue (arrow), preserved white matter, unique ectatic vessel (h@e x40 stain).

**Figure 11:**
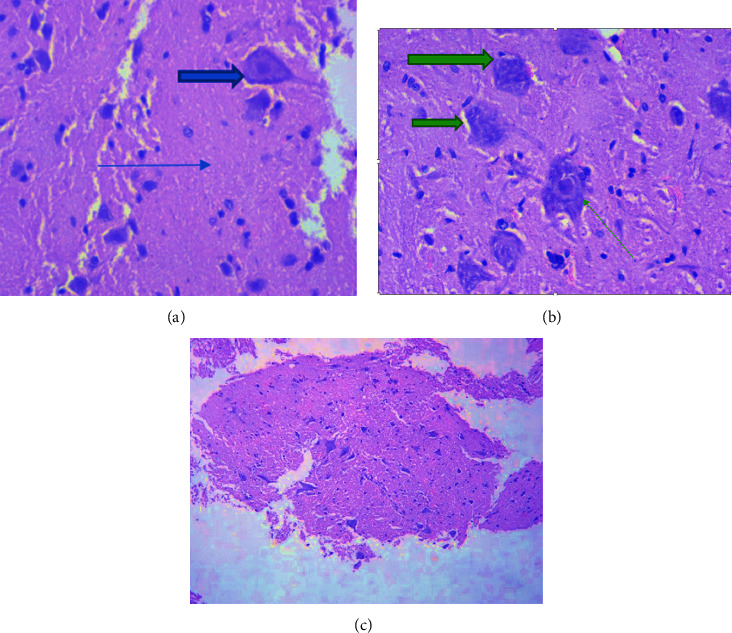
Morphology of rat spinal cord tissue: (a) (control) Preserved spinal cord tissue, visible motor neuron (thick arrow), white matter (arrow) without features. Hemotoxylin, eosin, x40. (b) (EAE) spinal cord tissue with H@E staining, ×40 magnification, wrinkling of motor neurons (arrow), karyolysis in individual neurons (bold arrow), white matter edema. (c) (Treatment) spinal cord tissue without features (staining h@e x40).

The results of histological examination showed that in the control group of rats, preserved brain tissue was observed, with visible cortical neurons, and the white matter showed no distinct features (Figure 10(a)). The morphology of the spinal cord tissue revealed preserved spinal cord tissue with visible motoneurons (thick arrow), and the white matter (arrow) showed no distinctive features (Figure 11(a)).

When examining the brain tissue of animals with experimental autoimmune encephalomyelitis (EAE), focal necrosis accompanied by brain edema, indicating brain damage, was observed (Figures 10(b) and 10(c)). In the spinal cord, dystrophic changes, including edema and wrinkling of neurons, were observed (Figure 11(b)).

Histological studies of brain and spinal cord specimens from rats receiving Cronassial in EAE revealed a predominance of oligodendroglial cells in the brain tissue (indicated by the arrow) (Figures 10(d) and 11(c)), preserved white matter, a unique ectatic vessel, and no distinctive features.

The results of the histological examination showed that the main morphological changes were observed exclusively in the group of animals with EAE in the white matter of the brain and spinal cord. These changes were characterized by cellular edema, focal necrosis, karyorrhexis, and karyolysis of motoneurons. Karyorrhexis involves the disintegration of the cell nucleus into parts and is one of the intermediate stages of necrobiosis, occurring after karyopyknosis and preceding karyolysis. During karyorrhexis, the cell nucleus's membrane is destroyed, and nucleic acids in the form of separate clumps penetrate into the cell's cytoplasm.

## 6. Discussion

The main difference of myelin from other membranes is its high lipid content, particularly glycosphingolipids. Lipids account for approximately 70–75% of the dry weight of the white matter of the central nervous system in mammals. The ratio of lipids to proteins is higher in the spinal cord. The high lipid content in the brain and its unique structure determine the nature of lipid peroxidation (LPO) processes within it. This disruption of lipid content leads to membrane destruction, inactivation of membrane-bound enzymes, and changes in permeability.

The lipid sheath of cellular and intracellular membranes serves two main functions: barrier and structural. Damage to the barrier function disrupts the regulation of intracellular processes and results in severe cellular dysfunction. The activity of membrane enzymes and receptors depends on the properties of the lipid phase of membranes, such as viscosity, surface charge, and polarity. Therefore, the activation of LPO processes, as found in our study, is considered a crucial element in the disruption of neuron and myelin membranes in multiple sclerosis. The administration of Cronassial partially normalized the investigated parameters.

Literature data indicate that gangliosides possess unique properties in vivo. Upon administration to the organism, they remain in the bloodstream for a relatively long time, are nontoxic, and in small amounts, they can penetrate the blood-brain barrier and become actively embedded in neuronal membranes. They promote the repair of damaged axons, offering significant therapeutic effects in cases of brain injury. It is known that gangliosides have a strong ability to interact with Ca++ ions. This enables them to maintain a constant concentration of free Ca++ at the presynaptic membrane, which ensures the functional activity of neural cells and their involvement in ionic transport processes [8].

Changes in intracellular calcium concentrations play a crucial role in initiating and regulating general and specialized cellular functions, such as proliferation, growth, gene expression, immune response, synaptic plasticity, metabolic control, and cell death. We hypothesize that one of the positive effects of Cronassial may be its influence on the normalization of calcium metabolism.

The hydroxyl radical most often causes protein aggregation, and in combination with the superoxide anion, it leads to fragmentation with the formation of low molecular weight fragments.

In fact, all amino acid residues of proteins are susceptible to oxidation, which alters their functions. Oxidation occurs in the sulfhydryl and amino groups of amino acids. As a consequence, cross-links are formed between proteins or between a protein and another molecule containing NH2 groups.

Literature data indicate that oxidative modification is considered one of the earliest markers of oxidative stress. The nature of oxidative protein modification depends on the type of reactive oxygen species (ROS) [17, 18]. The hydroxyl radical mainly causes protein aggregation, while in combination with the superoxide radical (O2), it leads to fragmentation. In the first case, covalently bound protein aggregates are formed in the form of high-molecular-weight complexes, such as dimers, trimers, and even tetramers. Oxidized proteins can be a contributing factor to the demyelination process, triggering a cascade of pathological events in multiple sclerosis. The increase in free radical products, such as lipids and proteins within the cell, disrupts the function of other subcellular components, including the nucleus and mitochondria.

As a result of our studies, a diffuse inflammatory-degenerative and atrophic process develops in the brain and spinal cord of experimental animals with autoimmune encephalomyelitis. This process includes edema of the white matter of the brain tissue, punctate focal necrosis, the formation of “reticular” tissue, and pericellular edema in pyramidal and oligodendroglial cells, all of which lead to damage to the myelin sheath and impaired conduction of nerve impulses.

Myelin membranes, formed by Schwann cells, envelop nerve fibers and serve as electrical insulators. This insulating layer covers most nerve fibers and significantly accelerates the propagation of electrical signals, allowing for the transmission of nerve impulses from one nerve cell to another. Damage to the myelin sheath triggers a cascade of inflammatory and immunological processes observed in multiple sclerosis.

Our studies have led to the conclusion that the formation and development of necrotic and inflammatory foci are observed in the brain and spinal cord tissues during EAE. The administration of the drug to animals with EAE preserved the white matter of the brain and spinal cord from inflammation, with a predominance of oligodendroglial cells, and increased the number of satellite cells.

Gangliosides have the ability to restore damaged axons following brain and spinal cord injuries [19, 20]. The protective properties of gangliosides are associated with their ability to normalize metabolic processes within the cell.

The results of our previous studies demonstrated that the administration of gangliosides as a therapeutic agent to rats with EAE had a protective effect on the development of oxidative processes within the mitochondrial fraction. Mitochondrial dysfunction and neuroinflammation with microglial activation can lead to neuronal death.

## 7. Conclusions

This study assessed the antioxidant properties of the commercial drug Cronassial and compared them to the well-known antioxidant alpha-tocopherol in EAE. The results of our research showed that small doses of the studied drugs are more effective than high doses and are more active than alpha-tocopherol in the pathology we studied. Our work elucidates and reveals the antioxidant properties of the ganglioside-containing drug Cronassial on the processes of free radical lipid oxidation and oxidative modification of proteins in the brains and spinal cords of rats with EAE. The results of our research demonstrate an increased formation of oxidative stress products, including peroxides, hydroperoxides, and oxidized proteins, in experimental animals, which is one of the factors contributing to myelin damage. The administration of a ganglioside-containing drug normalizes the effects of oxidative stress and inhibits the formation of reactive oxygen species. Thus, the data obtained indicate the neuroprotective and antioxidant effects of Cronassial when administered to animals with autoimmune encephalomyelitis.

Histological studies enable us to conclude that Cronassial partially restores damaged tissue structures in the brains and spinal cords of experimental animals.

## Figures and Tables

**Table 1:** The scores of clinical severity.

Number of point	Symptoms
0	No clinical signs; normal activity
1	Limp tail or hind limb weakness, but not both
2	Limp tail and hind limb weakness (*n* = 9 animals)
3	Partial hind limb paralysis (*n* = 9 animals)
4	Complete hind limb paralysis
5	Moribund state; death by EAE (*n* = 2 animals)

**Table 2:** Dynamics of physiological parameters of experimental animals (*n* = 26).

Physiological indicators, symptoms	Experimental groups			
	Control (*n* = 6)	EAE (*n* = 20)	EAE + Cronassial (*n* = 6) 20 mg/kg	EAE + *α*-tocopherol (*n* = 6) 20 mg/kg
Body weight (g)	210 ± 6.58	170 ± 9.38 ^*∗*^*p* = 0.027888	191 ± 4.58 ^*∗*^*p* = 0.026557 ^*∗∗*^*p* = 0.302920 ^*∗∗∗*^*p* = 0.475326	188 ± 5.23 ^*∗*^*p* = 0.027930 ^*∗∗*^*p* = 0.676232

*Note*. ^*∗*^*p*-compared with control, ^*∗∗*^*p*-compared treatment with pathology. ^*∗∗∗*^*p*-compared with alpha-tocopherol (vitamin E).

## Data Availability

The datasets used and/or analyzed during the current study are available from the corresponding author on reasonable request.

## Abbreviations

EAE: Experimental autoimmune encephalomyelitis
MS: Multiple sclerosis
DC: Diene conjugates
TC: Triene conjugates
HPO: Hydroperoxides
SB: Schiff bases
MDA: Malonic dialdehyde
LPO: Lipid peroxidation
ROS: Reactive oxygen species
CAF: Freund's adjuvant, complete
DNPH: Dinitrophenyl hydrazine.
